# Incorporating operational research in programmes funded by the Global Fund to Fight AIDS, Tuberculosis and Malaria in four sub-Saharan African countries

**DOI:** 10.1186/s12992-020-00599-8

**Published:** 2020-07-25

**Authors:** Salvador Camacho, Dermot Maher, Edward Mberu Kamau, Jasmina Saric, Luis Segura, Rony Zachariah, Kaspar Wyss

**Affiliations:** 1 Swiss Tropical and Public Health Institute, P.O. Box, CH-4002, Basel, Switzerland; 2 University of Basel, P.O. Box, CH-4003, Basel, Switzerland; 3 The Special Programme for Research and Training in Tropical Diseases, World Health Organization, P.O. Box, 1211, Geneva, Switzerland

**Keywords:** Implementation research, Operational research, Global Fund, Malaria, Tuberculosis, Sub-Saharan Africa

## Abstract

**Background:**

The current study builds upon a previous situation analysis of the extent to which grants from the Global Fund to Fight AIDS, Tuberculosis and Malaria (Global Fund) are being utilized to support operational research and implementation research (OR/IR) activities in recipient countries. The objective of this follow-up study was to identify approaches and pathways to implement an OR component into grants to the Global Fund, in four sub-Saharan African countries. Special focus was given to the Structured Operational Research and Training IniTiative (SORT IT).

**Methods:**

The conceptual framework was based on an analysis to identify elements supporting and blocking the integration of OR, called force field analysis, and a behavioural change assessment covering aspects such as opportunity, motivation, capability and triggers to do the integration. Data were collected through online surveys and stakeholder interviews both via telephone/online conference tools and in person in four countries with a high burden of malaria and tuberculosis. These countries were Ghana, Sierra Leone, the United Republic of Tanzania and Zimbabwe. The stakeholders included programme managers, implementation partners, representatives from international organisations, academic and governmental research institutions and other individuals involved in the countries’ needs assessment and National Strategic Plan development.

**Results:**

We identified opportunities to integrate OR into the countries’ programmes during the funding process, the country’s needs assessment being the most important one, including the need of OR-related capacity. Both the force field analysis and the behavioural change assessment showed that the necessary elements to integrate OR were present in the countries. Motivation, capability and efficiency were found to be a managerial value omnipresent across stakeholders. However, those elements were influenced by the tendency to favour tangible assets over any abstract ones, such as increasing organisational capacity in OR.

**Conclusions:**

In each of the countries assessed, there is potential to integrating OR into the programmes supported by the Global Fund. However, given the relative lack of OR-related capacity and skills encountered, a capacity strengthening tool, such as SORT IT, would be of benefit helping to identify and carry forward OR activities sustainably.

## Background

Three of the most devastating communicable diseases in human history, HIV/AIDS, tuberculosis (TB) and malaria, are being fought by affected low- and middle-income countries with the support of the Global Fund to Fight AIDS, Tuberculosis and Malaria (Global Fund), aiming at eventually ending those epidemics. The Global Fund disburses more than USD 4 billion a year to support programmes run by local experts in more than 100 countries, resulting in an estimated 32 million of lives saved [1]. It is the largest multilateral investor in health systems playing a leading role in global health by its contribution to financing the pathways towards universal health coverage in beneficiary countries. The Global Fund’s approach is multidimensional, shaping the global markets for medicines and technologies, increasing the resilience and sustainability of global health systems and strengthening human rights and gender equality within.

However, multiple challenges are present, especially on the path to disease elimination, including, emerging resistances along the malaria-transmission cycle (vector and agent), TB antimicrobial resistance [2, 3], weak implementation capacities, deficient drug supply systems, limited quality of care or fragmented approaches to health systems strengthening, urging the implementers of disease programmes to improve their efficiency. Operational research (OR) that is designed to increase both implementation efficiency and effectiveness provides evidence on elements that either enhance or impede the performance of established processes within, for instance, disease control activities [1, 4–7]. OR can therefore support programme managers and policy makers in optimizing and scaling up activities [1, 8, 9]. Also, in contrast to implementation research (IR) that may need more complex data of an intervention in pre-defined groups of patients, (e.g. a randomized control trial), OR often uses routine data to determine how interventions are translated into a benefit in the heterogeneous setting of routine care [4].

The Global Fund has recognized the value of OR and encourages its application by strengthening the capacity of recipient countries to collect high-quality data to maximize the impact of the GF-supported programmes [10]. For instance, OR has shown that gender and age inequities are important drivers of HIV and TB epidemics, the Global Fund has therefore included them as part of its Strategy 2017–2022 as key performance indicators, prompting its beneficiary countries to take actions to reduce those inequities [10]. Yet, OR and other evidence-based approaches are not being routinely embedded in control activities funded by the Global Fund despite the effort and advocacy from different stakeholders such as the Special Programme for Research and Training in Tropical Diseases (TDR) and local research organisations. The reasons for this have been previously explored by Kiefer and colleagues [11] revealing considerable variations from one country to another and between programmes with regards to the needs, demands, absorption capacity and funding for OR related to malaria and TB. The study by Kiefer and colleagues remarked the necessity of the involvement of national research coordination bodies, established research agendas and prioritizing human and technical research capacity to strengthen OR locally.

Building on the findings from Kiefer and colleagues [11], the Swiss Tropical and Public Health Institute (Swiss TPH), commissioned by TDR, undertook a follow-up investigation to find out if and how an OR tool could become an integral feature in the Global Fund grants. The Structured Operational Research and Training IniTiative (SORT IT), a global partnership coordinated by TDR and implemented with partners, was the main OR reference tool for this investigation of four recipient countries in sub-Saharan Africa. SORT IT is a training programme aimed at implementers with little or no prior research experience in which each participant learns practical skills of protocol writing, quality assurance, data collection and analysis, to finalize with a peer-reviewed journal publication [6]. The participants are also trained on the use of their findings to foster evidence informed decision-making in public health [12], as shown by the research by Prasad Tripathy and colleagues in 2018 [13] on how SORT IT Alumni’s work has influenced changes in national policies and practices in TB and HIV programs in Fiji, Moldova, India, and Myanmar, among other countries. For instance, in Myanmar, the national HIV programme routinely assessed creatinine clearance in patients taking Tenofovir, which is a costly procedure. However, based on the OR results by Kyaw and colleagues showing a low incidence of renal toxicity of the drug ([13, 14]), the national HIV programme adopted a low cost screening method for renal dysfunction, saving resources to be allocated elsewhere. The current study, therefore, builds upon Kiefer and colleagues’ previous situation analysis of the extent to which grants from the Global Fund to Fight AIDS, Tuberculosis and Malaria (Global Fund) are being utilized to support operational research and implementation research (OR/IR) activities in recipient countries [11]. The objective of this follow-up study was to identify approaches and pathways to implement an OR component into grants to the Global Fund, in four sub-Saharan African countries with a special focus on the SORT IT as a vehicle to increase OR capacity.

## Methods

Our study explores if there are the necessary conditions in the GF beneficiary countries to integrate OR elements into the programmes implementation, and what is the best way to do it. The conceptual framework was based on a behavioural change assessment and an analysis to identify elements supporting and blocking the integration of OR, namely force filed analysis. The assessment included the identification of opportunity, motivation, capability, and triggers within the specific country contexts as these elements are pointed out as necessary for a specific behaviour to be present [15, 16]. The force field analysis was used to assess the current situation within the selected countries because it has proven to be useful identifying both driving and blocking forces contributing to the actual behaviour equilibrium at a given time that are affecting a problem [17, 18]. The force field analysis also assists in the identification of the factors that can be dealt with to achieve a behaviour modification [19].

We used the SORT IT programme as the vehicle of OR because it represents a tried and tested approach to making use of routinely collected programme data to better understand how to improve programme performance [12, 20]. For instance, two of the most relevant papers published in the integration of OR to Global Fund grants are from Pakistan [21] and India [22]. These papers point out the effect of the SORT IT programme as an element that assists stakeholders coordination within the countries’ health system, which is a key element for successfully integration of OR into the disease programmes.

Four sub-Saharan African countries with high burden of malaria, TB or TB/HIV co-infection and that indicated interest in OR were included. The countries assessed in the current study were Ghana, Sierra Leone, the United Republic of Tanzania (Tanzania hereinafter) and Zimbabwe. The country selection was based on the following criteria: (i) high burden of malaria, TB and/or TB/HIV co-infection; (ii) having participated in the National Strategic Plan workshop organized by the Global Fund and the World Health Organization (WHO) in Hammamet, Tunisia in June 2019; (iii) having expressed interest in integrating OR into their national disease programmes funded or to be funded by GF grants; and (iv) Swiss TPH was not acting as a local fund agent in the country.

### The investigation was developed in two phases

#### Phase 1: desk review and process definition

We elaborate a depiction of the process that a country has to follow to implement a programme with funding from the Global Fund, to detect both opportunities for OR implementation and the most appropriate stakeholder to do so. The depiction consisted of reviews of publicly available documents on the Global Fund application process and stakeholder telephone consultations for, primarily, qualitative analysis. Representatives from the Global Fund, Local Fund Agent teams and technical assistants were consulted to crosscheck the depiction.

#### Phase 2: in-country data collection and analysis

This data collection phase included three main activities: (i) review of publicly available OR/IR documents relevant to countries that benefit from a collaboration with the Global Fund; (ii) country visits to carry semi-structured stakeholders interviews with key informants from different stakeholder groups (e.g. disease control programme managers, implementation partners, representatives from international organisations, members of academic and governmental research institutions and other relevant actors). Tanzania could not be visited therefore we conducted semi-structured interviews by telephone or using web-based conferencing tools. During the country visits, we also explored the awareness of the stakeholders of the SORT-IT programme or a similar one as a vehicle to integrate OR into their disease control strategies.

## Results

### Phase 1: desk review and process definition

**Fig. 1:**
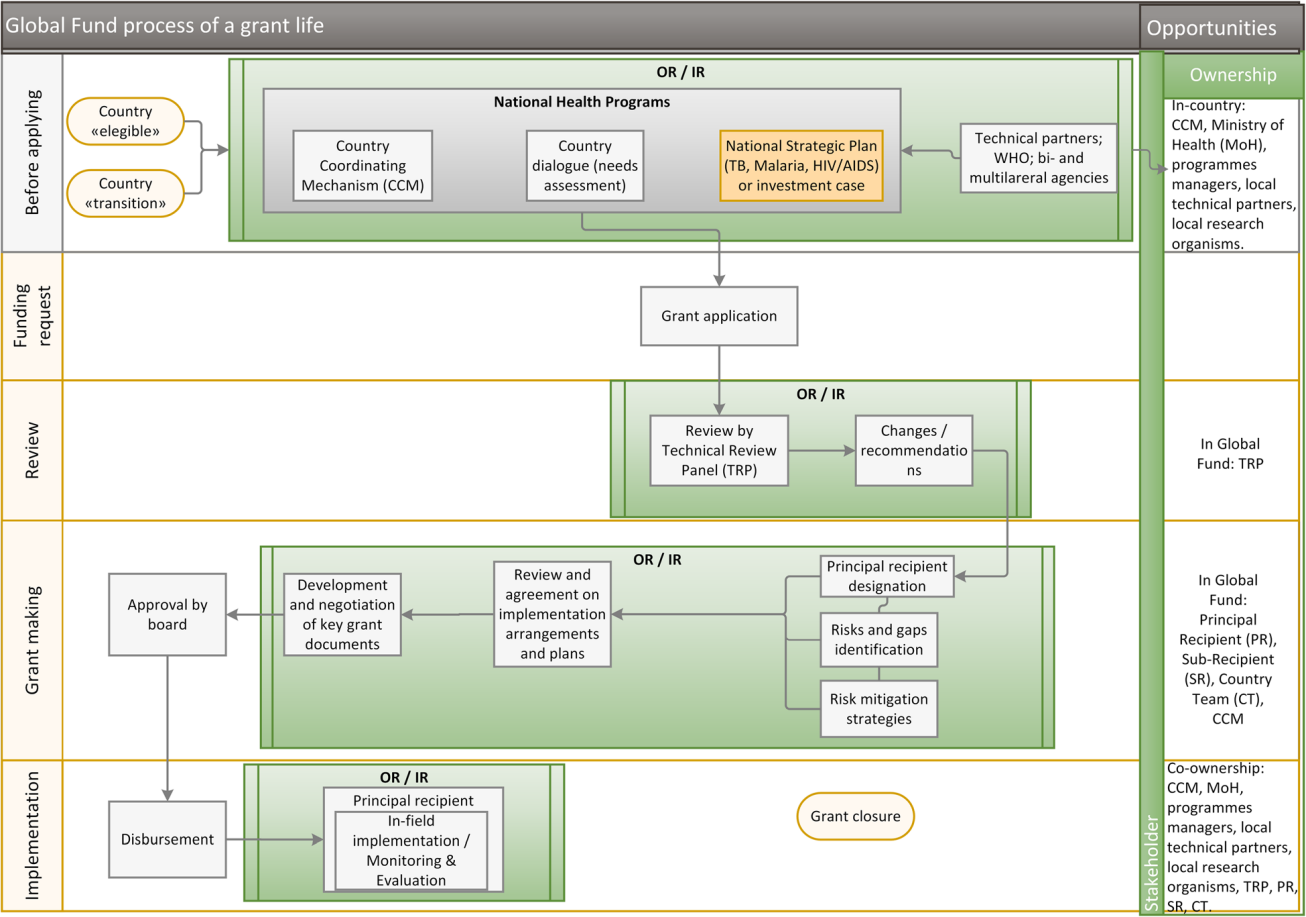
Ownership of opportunities for operational research implementation in a Global Fund grant life

Based on the relevant documents available, the process that a country has to follow to implement a programme with funding from the Global Fund was depicted. The ideal insertion point for OR implementation was identified to be within the phase prior to the submission of proposals to the Global Fund, especially during and after the country’s needs assessment. The ‘opportunity ownership’ was also assessed. It means that any action towards OR implementation has to be initiated by the owner of the opportunity, otherwise a conflict may appear. The process depiction, opportunity ownership and the OR specific insertion points throughout the grant life process are shown in Fig. 1. Twelve stakeholders were interviewed to crosscheck the accuracy of the process depiction, insertion points’ identification, and opportunity ownership.

### Country visits

Visits took place to Ghana, Sierra Leone and Zimbabwe, whereas Tanzania was assessed via telephone due to conflicting agendas between the study timelines of the mandate and the availability of the local stakeholders. The number of stakeholders interviewed were 21 in Ghana, 11 in Sierra Leone, 4 in Tanzania and 18 in Zimbabwe. During the country visits we identified the key stakeholders involved in the ‘prior to submission’ phase as the Ministry of Health (MoH), the Country Coordination Mechanism (CCM), the national control programme managers, local and international technical partners and research organizations, civil society representatives, and non-governmental organisations (NGOs). The involvement of the stakeholders varied from country to country suggesting that their involvement is not always fully assured. For instance, some interviewees mentioned that they “… develop the needs assessment in a participatory way through country dialogues”, whereas others mentioned that “the needs agenda is imposed by the Ministry of Health”.

### Role of stakeholders

The interviewed stakeholders perceived the CCM as a coordinating force whereas, the MoH was perceived as a multifaceted player steering implementation, advisory, technical assistance and needs assessment. Several stakeholders mentioned that CCM has the advantage “of not being under the governments’ payroll, so they can be objective and are not afraid of pointing out mistakes”. The Global Fund was identified as the most influential stakeholder able to provide normative guidance on the integration of OR in the countries.

### OR demand

We identified that the concept of OR is not homogenous among the countries nor the stakeholders. Regarding the SORT IT programme, only a few stakeholders knew the programme or similar tools to acquire OR skills.

Although efficiency on both programme implementation and use of resources was mentioned thoroughly across stakeholders and disease programmes, the donors, CCM or high-level government authorities did not articulate concrete demands in this regard. Some stakeholders mentioned that they “do not feel a strong pressure from Global Fund to optimize efficiency”, a perception that has been previously described in other studies [23–25]. The status quo of the programmes funded by the Global Fund did not commonly include OR.

### Behaviour change assessment

According to the COM-B [15] and Fogg’s behaviour models [16], a certain behaviour will manifest in a given moment when there is opportunity, a motivating factor, capability and triggers to do something.

#### Opportunity

The countries have several opportunities to integrate OR into the Global Fund funded programmes (Fig. 1). For instance, they can identify OR specific training in their needs assessment or they can add it to their National Strategic Programmes.

#### Motivation

The stakeholders of Global Fund grants – including programme implementers – have shown a positive attitude regarding any effort oriented to optimize the efficiency of Global Fund investments. However, the search for increased efficiency is a desire that may not currently materialize because the dialogue with the Global Fund and/or national stakeholders does not include metrics or incentives that reflect accurately the value for money [26–28]. Consequently, any effort oriented to increase efficiency has a lower priority when compared with the implementation of activities.

#### Capability

As pointed by Kiefer and colleagues [11], the current study confirmed that in each of the investigated countries there are several academic institutions (both national and international) and NGO’s present with research experience and expertise and the capacity to develop OR. However, partnerships to implement OR within the disease programmes are not flexible nor aligned to a national research agenda, except in a few cases. Given the weaknesses in the countries’ capabilities, the OR implementation partners within countries would benefit from capacity strengthening interventions such as SORT IT. For example:

- The acquired capacities will be available to the whole health system and not only to the programmes supported by the Global Fund;
- Being external to national disease control programme implementers, it may provide them with a more holistic and independent perspective about the performance of the programmes;
- By assembling OR capacities in as few single units as possible, instead of simultaneously in three programmes, the efforts to strengthen capacities in OR could be more efficient.

### Triggers

Programme reviews can act as triggers and they should be targeted by any initiative oriented to increase of OR/IR in Global Fund-supported grants.

Given that all the behavioural aspects are present in the studied countries, this suggests that the inaction to implement OR is due to either aspects other than behavioural ones or to a possible block of action from an influential stakeholder.

### Force-field analysis

**Fig. 2:**
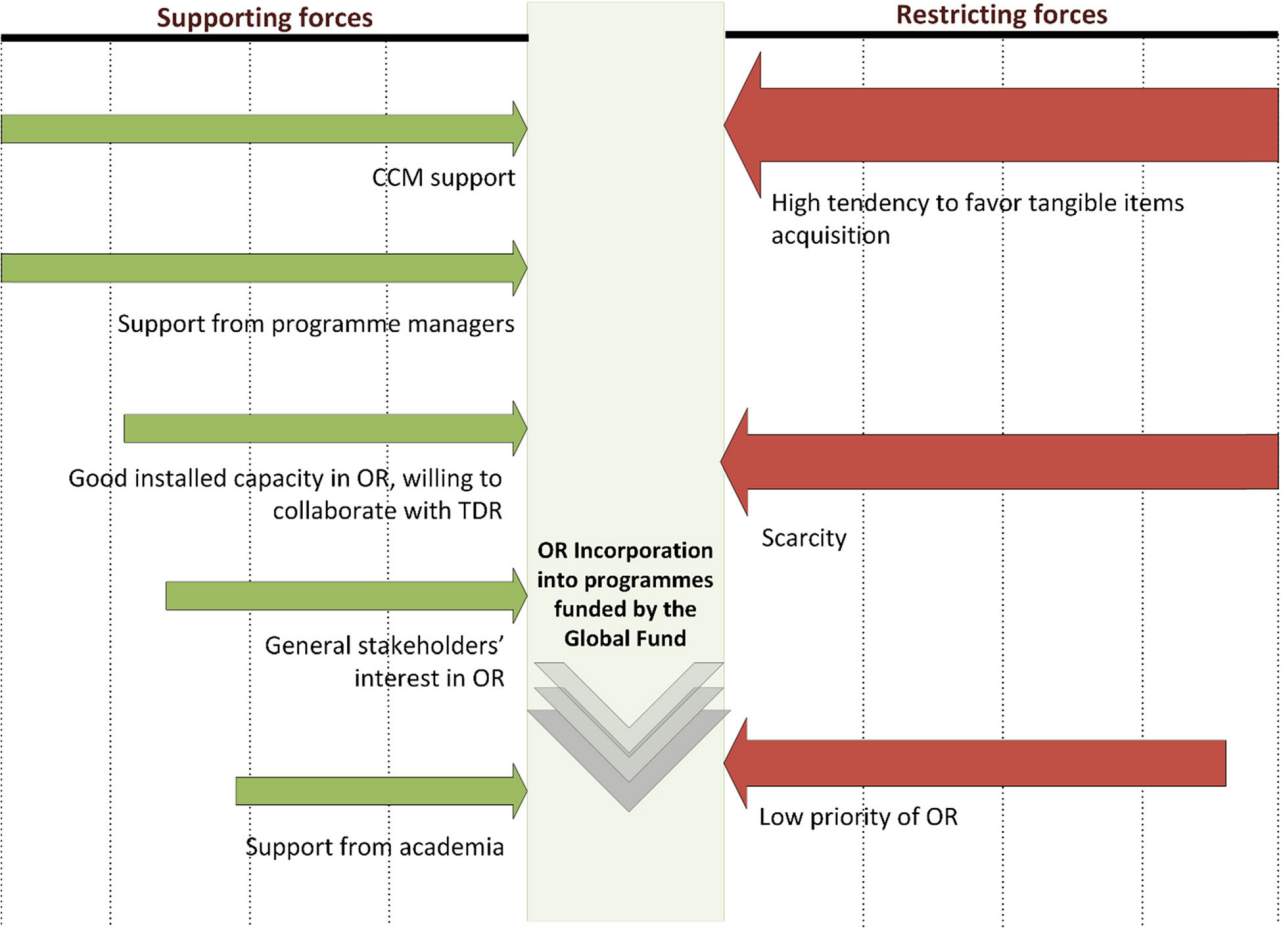
Force field analysis for operational research implementation in programmes funded by the Global Fund in Ghana, Sierra Leone, Tanzania, and Zimbabwe. Arrow sizes are reflecting the weight of each ‘force’CCM, Country Coordinating Mechanism; OR, operational research; SORT IT, Structured Operational Research and Training IniTiative; TDR, Special Programme for Research and Training in Tropical Diseases

Multiple forces favouring OR inclusion in Global Fund supported programmes were identified. The assistance from the different CCM members and their confidence to influence MoH seemed to be a key opportunity to be taken on board. In addition, civil society organizations, project managers, academia and international donors with presence in the countries (e.g. World Bank in Ghana) manifested their willingness to support OR implementation, as long as they were to be involved in the process and the trainings aim at sub-national level. On the other hand, forces opposing OR inclusion in Global Fund funded programmes were identified as follows: in face of the scarcity of funding, tangible goods (e.g. drugs or supplies) or well-known activities (e.g. training of health workers in case management) are commonly prioritized over OR. In addition, the uncertainty about the practical value of OR, does not generate the interest to search for extra funding to develop this niche activity. Any additional funding would be used, in a first instance, to procure the tangible goods, such as medicaments or bed nets, unless they are not specifically earmarked for OR (Fig. 2).

## Discussion

The interviewed stakeholders not only recognized the benefits of OR implementation but also manifested interest in integrating it into the relevant health programmes. The explored countries seem to have all the necessary elements to initiate the integration of OR into their health systems under the behaviour change assessment. However, no clear actions were identified towards this goal. Based on this insight, the behavioural aspects of OR adoption were taken into consideration assuming that a framework of incentives may be present preventing stakeholders to take any action. As mentioned before, the desire to enhance efficiency of programme implementation was found to be omnipresent across stakeholders. It is therefore surprising that those stakeholders barely recognized OR as an implementation tool, if at all.

Given that the main opportunity for countries to integrate OR elements into their programmes funded by GF is the country’s needs assessment, and the most influential stakeholder in this step is the MoH, we explored its role in this step. It is important to note that the country’s need assessment is the first step to elaborate the National Strategic Plan. Both the country’s need assessment and the MoH’s decisions seem to be greatly shaped by two factors: scarcity and tangibility. This favours the acquisition of tangible resources, e.g., drugs or bed nets, over non-tangible ones such as OR training which also results into a strong prioritization creating a perpetuating cycle that does not favour OR investments. Given the limited contributions of domestic funds to the country responses, the priorities of the national programmes are influenced by the requirements of the major donors. A change aiming to increase OR as a tool to increase value for money within the investments would require the involvement of both national and international stakeholders.

**Fig. 3:**
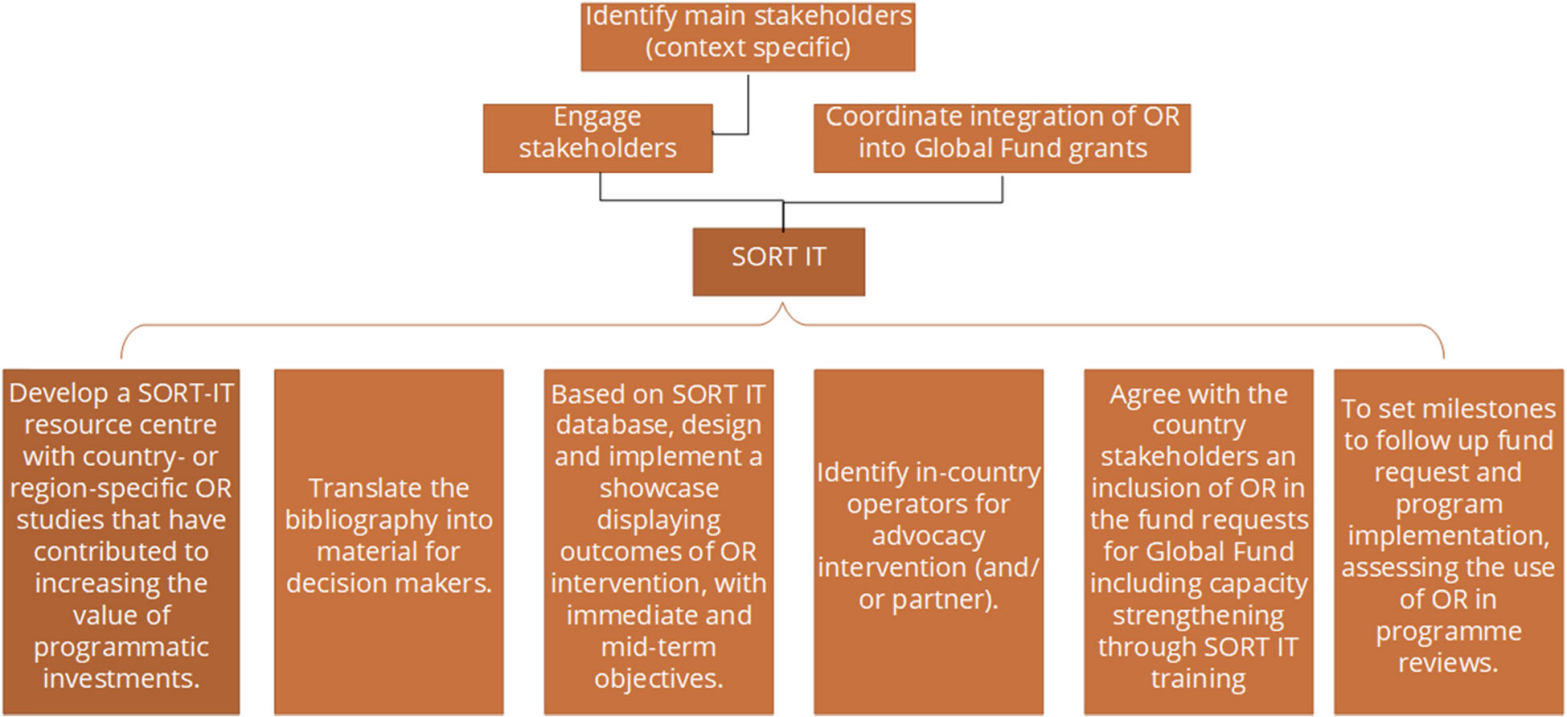
Crucial steps in the strategic plan towards integration of operational research into Global Fund grants in Ghana, Sierra Leone, Tanzania, and Zimbabwe. OR, operational research; Global Fund, The Global Fund to fight AIDS, Tuberculosis and Malaria; SORT IT, Structured Operational Research and Training IniTiative

The options to break the negative dynamics of scarcity and low prioritization of OR include donors earmarking funds to develop OR, either as part of the current commitments or as additional ones. Another option is to conduct a bottom-up approach with simultaneous sensitization among the in-country decision makers on the mid- and long-term benefits of OR with sound examples and data showing efficiency gains for national disease programmes. There are additional steps to consider in developing this option such as the coordination of the main stakeholders. For instance, there is a need for OR advocates to have strong links at country level so to engage with the national programmes thereby relying on the support of WHO country offices to effectively influence their approach to OR and their decisions in the country funding request. CCMs would be instrumental in this engagement as CCM could influence MoH decisions and strategy if a coordinated body of stakeholders backs it up. The crucial steps identified during our investigation are shown in Fig. 3.

Despite the differences in involvement, culture and local characteristics in the countries under review, the dynamics between the different stakeholders and the opportunities to integrate OR into GF grants were similar. This is probably due to the magnitude of the Global Fund financial support to health system and service development and derived structures, which homogenizes the particularities of Global Fund funded programmes among all countries. This opens the possibility that SORT IT could indeed work as a vehicle to integrate OR into the disease programmes funded by the Global Fund. Our recommendations to achieve this integration are presented in Table 1.

**Table 1:** Recommendations for engagement of stakeholders of national programmes to effectively influence their approach to OR and their decisions in the country fund request

● Develop a SORT-IT resource centre with real-life examples of OR increasing the value of programmatic investments, prioritizing examples coming from the same region.	
● Translate the bibliography into material for decision-makers (e.g. brochures, power point presentations, videos).	
● Design and implement a showcase displaying outcomes of OR intervention where SORT IT plays a major role, with immediate and mid-term objectives, e.g., personnel trained and efficiency increase, correspondently.	
● Identify in-country operators (and/or partners) for advocacy intervention. Country stakeholders beyond the MOH should be included to ensure sustainability of the process.	
● Agree with the country stakeholders on an inclusion of OR in the funding requests to the Global Fund (with support and technical assistance from TDR), including capacity strengthening through SORT IT training.	
● Set milestones to follow up funding request and programme implementation, assessing the use of OR in programme reviews.	

### Limitations

During the pre-visit phase, and besides all our efforts, we were unable to interview representatives from all the relevant stakeholders in each country on head quarter level and/or in-country, which may have affected representativeness. This limitation was reduced as much as possible by consulting official documents, e.g. position statements, whenever they were available. Another limitation may have been implicit bias (interview bias) where the respondent answers what he/she assumes to be the right answer and not necessarily what he/she honestly thinks. This bias was reduced as much as possible through the triangulation of the information.

## Conclusions

The necessary elements to integrate OR into the Global Fund funded programmes in Ghana, Sierra Leone, Tanzania and Zimbabwe seem to be in place. For instance, there is willingness from the stakeholders and opportunities to develop OR and integrate it into the Global Fund funded programmes. The SORT IT programme could be instrumental in the integration of OR into GF funded programmes by coordinating the CCM and other stakeholders to break the tendency of the MoH to favour the acquisition of tangible goods over OR training.

## Data Availability

All available data is presented in the descriptive tables of this manuscript. Source data can be obtained from the corresponding author.

## Abbreviations

CCM: Country Coordination Mechanism
CT: Country Team
GF: Global Fund
IR: Implementation research
MoH: Ministry of Health
NGO: Non-governmental organisations
NSP: National Strategic Plan
OR: Operational Research
PR: Principal Recipient
SORT IT: Structured Operational Research and Training Initiative
Swiss TPH: Swiss Tropical and Public Health Institute
TA: Technical Assistance
TDR: Special Programme for Research and Training in Tropical Diseases, hosted at the World Health Organization, and sponsored by the United Nations Children’s Fund, the United Nations Development Programme, the World Bank, and the World Health Organization.
TRP: Technical Review Panel
WHO: World Health Organization
